# 2201 A multi-stakeholder analysis on preparing future pediatricians to improve the mental health of children

**DOI:** 10.1017/cts.2018.274

**Published:** 2018-11-21

**Authors:** Cori M. Green, John Walkup, William Trochim

**Affiliations:** New York Presbyterian Hospital, Weill Cornell Medicine

## Abstract

OBJECTIVES/SPECIFIC AIMS: (1) Develop a concept map of ideas from diverse stakeholders on how to best improve training programs. (2) Assess the degree of consensus amongst stakeholders regarding importance and feasibility. (3) Identify which ideas are both important and feasible to inform policy and curricular interventions. METHODS/STUDY POPULATION: Concept mapping is a 4 step approach to data gathering and analysis. (1) Stakeholders [pediatricians (peds), MH professionals (MHPs), trainees, parents] were recruited to brainstorm ideas in response to this prompt: “To prepare future pediatricians for their role in caring for children and adolescents with mental and behavioral health conditions, residency training needs to...”. (2) Content analysis was used to edit and synthesize ideas. (3) A subgroup of stakeholders sorted ideas into groups and rated for importance and feasibility. (4) A large group of anonymous participants rated ideas for importance and feasibility. Multidimensional scaling and hierarchical cluster analysis grouped ideas into clusters. Average importance and feasibility were calculated for each cluster and were compared statistically in each cluster and between subgroups. Bivariate plots were created to show the relative importance and feasibility of each idea. The “Go-Zone” is where statements are feasible and important and can drive action planning. RESULTS/ANTICIPATED RESULTS: Content analysis was applied to 497 ideas resulting in 99 that were sorted by 40 stakeholders and resulted in 7 clusters: Modalities, Prioritization of MH, Systems-Based, Self-Awareness/Relationship Building, Clinical Assessment, Treatment, and Diagnosis Specific Skills. In total, 216 participants rated statements for importance, 209 for feasibility: 17% MHPs, 82% peds, 55% trainees. There was little correlation between importance and feasibility for each cluster. Compared with peds, MHPs rated Modalities, and Prioritization of MH higher in importance and Prioritization of MH as more feasible, but Treatment less feasible. Trainees rated 5 of 7 clusters higher in importance and all clusters more feasible than established practitioners. DISCUSSION/SIGNIFICANCE OF IMPACT: Statements deemed feasible and important should drive policy changes and curricular development. Innovation is needed to make important ideas more feasible. Differences between importance and feasibility in each cluster and between stakeholders need to be addressed to help training programs evolve.

